# Effects of non-tuberculous mycobacteria on BCG vaccine efficacy: A narrative review

**DOI:** 10.1016/j.jctube.2024.100451

**Published:** 2024-05-04

**Authors:** Fatemeh Ghasemi, Jalil Kardan-Yamchi, Mohsen Heidary, Morteza Karami-Zarandi, Sousan Akrami, Abbas Maleki, Saeed Khoshnood, Hossein Kazemian

**Affiliations:** a Division of Microbiology, Department of Pathobiology, School of Public Health, Tehran University of Medical Sciences, Tehran, Iran; b Quality Control and Screening Management Office, Deputy of Technical and New Technologies, Iranian Blood Transfusion Organization, Tehran, Iran; c Cellular and Molecular Research Center, Sabzevar University of Medical Sciences, Sabzevar, Iran; d Department of Microbiology, Faculty of Medicine, Zanjan University of Medical Sciences, Zanjan, Iran; e Department of Microbiology, Faculty of Medicine, Tehran University of Medical Sciences, Tehran, Iran; f Clinical Microbiology Research Center, Ilam University of Medical Sciences, Ilam, Iran; g Department of Microbiology, Faculty of Medicine, Ilam University of Medical Sciences, Ilam, Iran

**Keywords:** BCG, Non-tuberculous mycobacteria, NTM, Tuberculosis, Vaccine, TB

## Abstract

The *Mycobacterium tuberculosis* bacterial pathogen is responsible for the ongoing global tuberculosis (TB) epidemic. Bacille Calmette-Guérin (BCG), the only currently approved TB vaccine, is successful in preventing disseminated disease in newborns. However, it has a variable efficacy against pulmonary TB in adults. This protective effect of the vaccine varies greatly among different populations and geographical areas, which the increased exposure of particular populations to non-tuberculous mycobacteria (NTM) is considered as one of the reasons for this issue. Numerous studies have shown that exposure to NTM species causes the host immune system to be improperly primed. It has also been suggested that NTM species may be blamed for reduction in BCG vaccine effectiveness against *M. tuberculosis*. The increased exposure of certain populations to NTM has diverse effects on BCG efficacy. Moreover, the exposure to NTM can induce opposite effects on BCG efficacy depending on the NTM exposure route and survivability. A detailed understanding of the impact of NTM exposure on the efficacy of the BCG vaccine is essential for ongoing efforts to develop new TB vaccines as it may ultimately be a crucial success factor. The aim of this study was to review the findings of the studies focusing on the effects of NTM on BCG vaccine efficacy in animal models.

## 1 Introduction

Bacillus Calmette-Guérin (BCG), an attenuated strain of *Mycobacterium bovis*, is the only licensed vaccine against human tuberculosis (TB) [1], [2]. TB is still one of the most significant health issues worldwide, despite the widespread use of BCG vaccine. In 2022, an estimated 10.6 million people, including 1.3 million children, 3.5 million women, and 5.8 million men, fell ill with TB, and about 1.3 million people died from this infectious disease. TB is present worldwide and affects all age groups [3]. Animal model studies have suggested that different strains of BCG, including Pasteur 1173 P2, Danish 1331, Glaxo 1077 (derived from the Danish strain), Tokyo 172-1, Russian BCG-I, and Moreau RDJ, have a highly variable protective effect. Vaccines containing the Glaxo 1077, Tokyo 172-1, or Moreau RDJ strains have been proven to cause fewer side effects than those contain other strains. The number of live bacteria in a single dose is affected by the strain and can range from 50,000 to 3,000,000 [4]. The highest and lowest levels of virulence were observed in BCG strains from the DU2 group IV (BCG-Phipps, BCG-Frappier, BCG-Pasteur, and BCG-Tice) and DU2 group II (BCG-Sweden and BCG-Birkhaug) respectively. Strain-specific genomic DNA duplications and deletions may be responsible for this difference in virulence. It is speculated that more virulent BCG strains have more efficiency in protection against *Mycobacterium tuberculosis* (*M. tuberculosis*) challenge [5]. A recent study has evaluated differences between varying strains of BCG and conferred no difference in progression-free, recurrence-free, cancer-specific, and overall survival after using RIVM, TICE, and Moreau strains. Moreover, strain change during the therapy was associated with the high risk of mild to severe toxicity [6]. The genetic diversity of BCG vaccine strains has been proven to influence the immunogenicity, pathogenicity, and survivability of a vaccine strain, which all are expected to alter protection against TB caused by BCG vaccination [7].

BCG has shown a variable protective effect against non-tuberculous mycobacteria (NTM) infections [8], [9]. In this regard, various strategies have been implemented to improve or enhance the protective efficacy of BCG [10], [11], [12]. The efficacy of BCG vaccines can be affected by genetic differences among human populations [1], [13], [14]. An earlier systematic review has also reported that the effectiveness of BCG vaccination varies between different populations, diseases, age group, and BCG strain used [15]. The presence of NTM in the environment is another factor involved in the variability of BCG efficacy. It has been proposed that exposure to NTM can cause cross-reactive immune responses, resulting in the inhibition of BCG activity or replication and/or reduction of specific immune responses, e.g. IFN-γ, induced by BCG against TB [14]. In a meta-analysis, it was found that the high efficacy of BCG is related to the absence of exposure to NTM and pre-existing infection will hinder the protective efficiency of the vaccine [16]. In animal models, NTM share cross-reactive antigens that can give rise to significant protection against *M. tuberculosis* [17]. Notwithstanding widespread debate on the interventional effects of NTM on the efficacy of BCG vaccine, there are still controversial views in this matter [18], [19], [20], [21]. To find a comprehensive answer to this debatable issue, the present study was conducted to review published literatures assessing the effects of NTM on BCG vaccine protection against TB.

## 2 Interference of *Mycobacterium avium*-*intracellulare* (MAI) with BCG

BCG is used in TB-endemic regions to prevent severe forms of TB in children and adolescents and develops cross-protective T cell immunity against MAI [22]. Among the mycobacterial species, MAI was the most extensively studied species reviewed in 14 papers. These studies were performed on three different animal models, including mice (nine papers) guinea pig (three papers) and calve (two papers). Animal sensitization with MAI was carried out before (pre-sensitization) and after (post-vaccination) BCG vaccination in 12 and 2 studies, respectively. Among the 14 studies reviewed, two studies administered both pre-sensitization and post-vaccination. Sensitization and vaccination routes used in the reviewed studies are depicted in Table 1. According to the results of investigations included, eight studies reported that MAI has interference with the BCG protection [17], [21], [2], [1], [23], [24], [25], [26]. However, six other studies showed that sensitization with this mycobacterial species did not interfere with the protective efficacy of the vaccine [14], [27], [28], [29], [30], [20]. The predominant experiments used in most of these studies were bacteriological and histopathological tests. In two studies, exposure to NTM induced positive and negative effects on BCG efficiency depending on the mode of sensitization. In one study, pre-exposure to MAI decreased the efficacy of BCG vaccine [31]; however, another study reported opposite result [32]. There was no study indicating the positive effect of MAI on BCG vaccine efficacy, except for one study in which exposure to killed MAI administered by a systemic route enhanced BCG efficacy [1]. Price et al., indicated that exposure to MAI did not interfere with BCG vaccine efficacy via the pulmonary route [2]. Most studies on mouse models, but not studies performed on guinea pig and calve, represented a reducing effect on vaccine efficacy. The variables of reviewed studies are detailed in Table 1.

**Table 1:** Characteristics of included studies.

**Mycobacterial species**	**Sensitization**	**Animal model**	**Exposure route**	**Vaccination route**	**Effect in BCG vaccine**	**BCG strain**	**Alive or inactivated NTM**	**Author, publication year**	**Ref.**
			**Time elapsed:**	**Time elapsed:**					
			6 weeks	6 weeks					
*M. kansasii*	Pre	Mice	SC	IV	Not interfered	*M. bovis* BCG Pasteur	Heat-killed	Orme et al., 1983	[38]
			40 days	40 days					
*M. kansasii*	Pre	Mice	IV, SC	SC	Not interfered	*M. bovis* BCG Pasteur	Live	Orme et al., 1986	[29]
*M. avium*									
			70 days, 40 days	70 days					
*M. kansasii, M. simiae*, *M. avium*, MSC	Pre	Mice	IN	IV	Not interfered	*M. bovis* BCG	Live	Orme et al., 1984	[20]
			30 days	60 days					
*M. avium*	Pre	Mice	ID, oral	ID, pulmonary	Decreased	*M. bovis* BCG Pasteur	Killed	Price et al.,2016	[2]
			4 weeks	1 week					
*M. avium*	Post	Mice	IP, oral	SC	IP: Improved	BCG Pasteur	IP: killed *M. avium*	Poyntz et al., 2014	[1]
			12 months, 28 weeks	−	Oral: Decreased		Oral: live *M. avium*		
*M. avium*	Pre	Mice	IP	SC	Decreased	*M. bovis* BCG	Autoclaved	Yang et al., 2012	[23]
			48 h	2 h					
*M. avium*	Pre	Calve	SC	SC	Decreased	*M. bovis* BCG	Live	Thom et al., 2008	[14]
			12 weeks	2 weeks					
*M. avium*	Pre	Mice	Oral	SC	Decreased	*M. bovis* BCG	Live	Young et al., 2007	[21]
			8 weeks	8,16,24					
*M. avium*	Post	Mice	Oral	SC	Decreased	*M. bovis* BCG Pasteur	Live	Flaherty et al., 2006	[24]
			16 weeks	35 days					
*M. avium*	Pre	Calve	SC	SC	Improved	*M. bovis* BCG	Live	Howard et al/ 2002	[28]
			12 weeks	24 weeks					
*M. avium* *M. vaccae*, MSC	Pre	Mice	SC	SC	Improved	recombinant BCG strain expressing RD1 antigens	Live	Demangel et al., 2005	[25]
			4 weeks	2 weeks	(*M. avium > M. scrofulaceum,* or *M. vaccae)*				
MAI	Pre, post	Guinea pig	Oral, ID	ID	Improved	BCG strain no. 1331	Live	Edwards et al., 1982	[32]
			2 weeks	4 and 24 h					
			6 weeks	3 weeks					
Cocktail (MAI*,* MSC, *M. vaccae*)	Pre	Mice	SC	SC	Decreased	BCG Danish 1331	Live	Brandt et al., 2002	[47]
			6 weeks	6 weeks					
MAI*,* MSC	Pre	Mice	IV	IV	Improved	*M. bovis* BCG	Live	Lozes et al/ 1997	[17]
			19 days	4 weeks					
MAI*,* MSC	Pre, post	Guinea pig	SC	SC	Not interfered	BCG Glaxo	Live	Herbert et al., 1994	[31]
			6 weeks	6 weeks					
MAI, *M. simiae*	Pre	Guinea pig	ID	ID	Not interfered	BCG-Copenhagen	Live	Smith et al., 1985	[30]
			6 weeks	6 weeks					
*M. vaccae*	Pre	Mice	Oral	SC	Improved	BCG Glaxo	heat-killed	Brown et al., 1985	[39]
			3 weeks, 27 days, or 54 days	50 days					
			9 months	3 months					
			3 weeks	1 weeks					
*M. chelonae*	Pre	Mice	IN, IP	IN	Decreased	*M. bovis* BCG Pasteur	Heat-killed	Ho et al., 2010	[43]
			3 weeks	5 days					

**Abbreviations:** ID: Intradermal; IP: Intraperitoneal; SC: Subcutaneous; IV: Intravenous; MSC: *M. scrofulaceum;* MAI: *M. avium-intracellulare*; Ref: reference.

## 3 Interference of *Mycobacterium scrofulaceum* with BCG

Findings have shown that the presence of *M. scrofulaceum* in the environment may interfere with the protective effectiveness of BCG vaccine against TB in genetically susceptible people. Four studies examined BCG vaccine protection against *M. scrofulaceum* [17], [20], [25], [31]. Pre-exposure of mice to *M. scrofulaceum* in three studies indicated no interference, decrease, or increase in immune responses induced by BCG vaccination. However, the last study on guinea pig displayed decreased vaccine protection against this bacterium before but not after vaccine administration. This reduction behavior depends on the time interval between sensitization and vaccination and also on multiple exposures to NTM. During the early stages of vaccination, no interference was observed in the immune responses due to BCG, but at later stages of vaccination, the immune barriers may not be intact in the tested animal models. Moreover, multiple exposures to this NTM resulted in a waned protection than a single exposure [31].

## 4 Interference of *Mycobacterium kansasii* with BCG

The transcriptome of infected macrophages analyzed in a recent study suggested that genes encoding inducers of Type I IFN responses, such as cytosolic DNA sensors, are less expressed in *M. kansasii*-infected macrophages than BCG-infected macrophages [33]. *M*. *kansasii* causes lung disease, a clinically similar symptom to TB, and is inherently resistant to the first-line antibiotics, such as pyrazinamide [34]. While *M. kansasii* infections induce minor symptoms, it is known that more severe symptoms can be developed when the infection co-occurs with other conditions such as inflammatory pseudotumor [35]. In an animal study investigating the impact of BCG strain on immunodiagnostic tests, exposure to *M. kansasii* induced sensitization without disease [36]. In another study on the experimental challenge of cattle with *M. kansasii*, BCG strain had varied effects on serological TB tests [37]. Several studies have also assayed variations in vaccine performance in a mouse model after sensitization with *M. kansasii* [29], [20], [38]. No interference of this species was reported in different administration routes of NTM and prior to BCG vaccination. Orme and Collins signified that despite infection with *M. kansasii*, mice intravenously vaccinated with *M. tuberculosis* showed resistance [20]. Also, no effect of intravenous or subcutaneous infection with *M. kansasii* has been demonstrated in another study conducted by Orme et al [29].

## 5 Interference of *Mycobacterium vaccae* with BCG

Interference of *M. vaccae* with BCG vaccine efficacy was evaluated in two studies. One of the studies disclosed that *M. vaccae* is phylogenetically distinct from BCG. This evidence was indicated by immune responses in mice exposed to this environmental mycobacterium. Moreover, exposure to *M. vaccae* directly affected BCG vaccination and showed low protection against TB [25]. In another study, mice were given *M. vaccae* in their drinking water for 21 days immediately, 27 days, or 54 days before they were injected subcutaneously with BCG. The results showed that, oral administration of *M. vaccae* can enhanced or interfered with BCG sensitization, depending on the in vivo exposure time to *M. vaccae* before the injection of BCG and the dose of the mycobacterial challenge in vitro [39]. *M. vaccae* National Collection of Type Cultures (NCTC) 11,659 has lately attracted interest for the prevention and treatment of different conditions, including TB. However, its effective use needs a thorough knowledge of this environmental saprophytic bacterium [40].

## 6 Interference of *Mycobacterium chelonae* with BCG

*M. chelonae* is mostly found in water and soil and on poorly sterilized medical equipment. This bacterium has been defined as pathogen of fish, but it has been found in mice, snakes, turtles, cattle, pigs, cats, and dogs. *M. chelonae* can cause infection in different organs, such as lungs, lymphatic system, skin, and soft tissue and is resistant to antibiotics more than other rapidly growing mycobacteria [41]. *M. chelonae* has emerged as common etiology of port site infections after laparoscopic surgeries due to breach in sterilization protocols [42]. In two studies, Ho et al., reviewed pre-sensitization of mice with *M. chelonae*. Both studies illustrated that prior exposure to *M. chelonae* has no interference with live BCG in an intranasal or intraperitoneal route. Moreover, prior exposure to *M. chelonae* led to a significant increase in the IFN-γ-producing CD4^+^ cells and was correlated with several folds of reductions in lung BCG counts. *M. chelonae* sensitization also resulted in CD4^+^-mediated cytotoxicity against BCG and induced CD4^+^CD25^+^ regulatory T cells with suppressive function against BCG-mediated immunity [43], [44].

## 7 Interference of *Mycobacterium simiae* with BCG

*M. simiae* is a slowly growing mycobacterium that was first obtained from Indian Rhesus monkeys. Infection caused by this bacterium seems to be restricted to certain geographical regions, including the Middle East countries, Western Europe, Southwestern United States, and Cuba. *M. simiae* infection has a pooled prevalence of 25 % among NTMs in Iran [45]. BCG's ineffectiveness against *M. simiae* may be due to the lacks most of cross-reactive antigens against this NTM [34]. *M. simiae* lowers immunizing ability of BCG, most likely by exerting an immunosuppressive effect [46]. The interventional effect of *M. simiae* on the efficacy of BCG vaccine was investigated in two studies on guinea pig and mice. In two studies conducted by Smith et al., and Orme et al., it was found that prior infection with *M. simiae* did not have any impact on the induction of acquired immunity to BCG [30], [20].

## 8 NTM cocktail

The cocktail of three species, including *M. scrofulaceum, M. avium*, and *M. vaccae*, were investigated in two different studies. Subcutaneous treatment of this cocktail resulted in low BCG efficacy in a mouse model. This phenomenon was observed due to the presence of *M. avium* in the cocktail. Bacterial numbers in the organs of naive and sensitized mice after vaccination and *M. tuberculosis* challenge demonstrated that the multiplication of live BCG was blocked by *M. avium* [25], [47].

## 9 Possible mechanisms of the interference

A theory suggests that BCG effectiveness is associated with exposure to NTM found in the environment [14]. Mycobacterial species have a close genetic relationship and share various similar antigens [44], [48], resulting in an induced immune response that interferes with the generation of BCG-specific immunity or replication of *M. bovis* BCG [14]. Experimental studies have represented controversial observations regarding BCG protection against TB. These investigations have shown that BCG protection could be reduced or increased following exposure to environmental mycobacteria [1], [14].

In the present review, we included animal model experiments indicating the cross-immunity of BCG vaccine against NTM. Sensitization by *M. avium* can lead to the failure of BCG to induce any protection against TB, owing to the clearance of BCG by antimycobacterial immune responses. A reason that NTM have no significant reduction in vaccine protection is a type of tested animal model, i.e. C57BL/6 mice, which can be more resistant to TB [25]. Furthermore, the route of vaccination is an important factor in vaccine efficacy. It has been reported that after intradermal BCG vaccination in mice exposed to *M. avium*, a significant decrease occurs in the proinflammatory cytokine IFN-γ and an increase in the regulatory T cells and immunosuppressive cytokine of IL-10. Unlike intradermal BCG immunization, pulmonary BCG vaccination shows protectivity against TB, regardless of previous *M. avium* exposure [2]. Moreover, another paper reported that exposure to NTM can induce adverse effects on BCG efficacy, depending on the route of exposure and viability of NTM. BCG effectiveness can be enhanced by exposure to killed *M. avium* administered by a systemic route, leading to T helper 1 and T helper 17 responses and is associated with increased protection. Conversely, BCG efficacy can be declined by exposure to live *M. avium* administered by the oral route, which results in T helper 2 response [1].

Pre-exposure to NTM dampens the immune response against M. tuberculosis, and this dampening is associated with the downregulation of cell response to IFN-γ stimulation and the upregulation of antibody responses, which are characteristics of a switch to a type 2 immune response [21], [23]. Interestingly, the adverse effect of NTM on BCG efficacy depends critically on the extent of cross-recognition of antigens shared with the BCG vaccine. For instance, *M. vaccae* as a fast-growing mycobacterium is phylogenetically more distant from BCG than the slow-growing mycobacteria such as *M. scrofulaceum* and *M. avium*. However, these genetically connections innately causes immune responses that cross-react with *M. tuberculosis* Ag85B expressed in all above-mentioned environmental mycobacteria [25].

Virtually all studies reviewed in this study divulged that prior exposure to NTM species had adverse effects on BCG or showed no significant reduction in vaccine protection. It has also been demonstrated that BCG was more affected than recombinant BCG containing RD1 antigens, when exposed to environmental mycobacteria. This finding is dependent on the extent of cross-recognition of antigens shared with BCG [25]. BCG efficacy has been shown to have low level of protection in geographic regions with high burden of NTM [1].

## 10 Future perspectives

Exposure to NTM is probably a contributory factor in the reduction of BCG efficacy. Considering the model of exposure used, exposure to NTM can affect BCG mediated protection. Humans are often exposed to NTM species through varying routes of infection and also via living and nonliving bacteria. Therefore, it is essential to identify exposures that reduce the protection efficacy of BCG and to elucidate the immunological mechanisms that alter the protective response induced by BCG. Identification of factors involved in the variable efficacy of BCG could help to the predict to what extent the efficacy of a novel TB vaccine candidate will be influenced [1]. There are many gaps and differences between humans and animal models regarding immunological response to BCG vaccination. Most studies investigated the effects of the BCG vaccine on NTM species have used mice models. Thus, development of new models, such as nonhuman primate and guinea pigs, seems to be crucial. Using human studies and animal models that most closely resemble the target population, humans, is undoubtedly the wisest course of action. In most cases, mouse models have led us down a path of false information. The guinea pig and nonhuman primate models are key models that can be developed and mimic humans. Overall, researchers and scientists are required to use models that represent NTM infection and disease and also to provide data on how BCG vaccination and NTM persistence could influence the human host [49].

## 11 Conclusion

Worldwide, BCG vaccination programs are routinely used to guard infants against TB. For unknown causes, the effectiveness of BCG vaccine in preventing TB has varied greatly. The interaction of NTM with the immune system has been proposed as a cause for the variation in protection provided by BCG. However, the precise mechanism has so far not been clarified. The increased exposure of certain populations to NTM has diverse effects on BCG efficacy. Moreover, the exposure to NTM can induce opposite effects on BCG efficacy depending on the NTM exposure route and survivability. A detailed understanding of the impact of NTM exposure on the effectiveness of the BCG vaccine is necessary for ongoing efforts to develop new TB vaccines. Additional research is needed to longitudinally follow how NTM exposure affects host-protective anti-TB immunity produced by the BCG vaccine.

## Funding

Not applicable.

## Ethical Statement

Not applicable.

## CRediT authorship contribution statement

**Fatemeh Ghasemi:** Writing – review & editing, Writing – original draft. **Jalil Kardan-Yamchi:** Conceptualization. **Mohsen Heidary:** Writing – original draft. **Morteza Karami-Zarandi:** Writing – original draft. **Sousan Akrami:** Writing – original draft. **Abbas Maleki:** Writing – original draft. **Saeed Khoshnood:** Writing – review & editing, Writing – original draft. **Hossein Kazemian:** Supervision, Methodology, Investigation, Conceptualization.

## Declaration of competing interest

The authors declare that they have no known competing financial interests or personal relationships that could have appeared to influence the work reported in this paper.
